# A mouse GITRl fusion protein drives T cell activation and antitumor activity in preclinical mouse models of cancer

**DOI:** 10.1186/2051-1426-3-S2-P191

**Published:** 2015-11-04

**Authors:** Rebecca Leyland, Amanda Watkins, Lisa Bamber, Natalie Tigue, Lesley Young, Michelle Morrow, Scott Hammond, Robert Wilkinson, Ross Stewart

**Affiliations:** 1 MedImmune, Cambridge, UK; 2 MedImmune, Gaithersburg, MD, USA

## 

GITR is a member of the TNFR superfamily of proteins and is expressed on resting regulatory T cells and on other T cells, and NK cells, following activation. Signals through GITR have been shown to drive increased T cell activity and reduced regulatory T cell function. In order to explore the potential of therapeutically targeting GITR in a cancer setting, we generated a mouse GITRL fusion protein (mGITRL FP) consisting of the extracellular domain of mGITRL linked to a structural domain and an IgG Fc domain. The antitumor activity and pharmacodynamic effects of this molecule were then explored in the colorectal syngeneic model of cancer (CT26).

Treatment of mice with mGITRL FP mediated anti-tumor activity that was dependent on isotype and exposure. The anti-tumour activity could be attributed at least in part to the increased activation and proliferation status of CD8+ and CD4+ T cells, as evidenced by increases in Ki67 expression, ICOS upregulation and increased cytokine secretion of these cells. Intratumourally we observed a significant decrease in the frequency of CD4+ T cells (including T regs), but a corresponding increase in cytotoxic CD8+ T cells.

OX40 is another member of the TNFR superfamily, that has similar expression and functions to GITR. In order to better understand the potential differences between targeting of these two pathways, the activity and pharmacodynamic effects of the mGITRL FP were additionally compared and contrasted to those of a mOX40L FP and the observed differences will be discussed.

